# Rheumatology specialist care in Europe: workforce trends and regional variations from a UEMS survey (European Union of medical specialists)

**DOI:** 10.3389/fmed.2026.1786042

**Published:** 2026-02-24

**Authors:** Eva Rath, Rudolf Puchner, Christian Dejaco, Juergen Wollenhaupt

**Affiliations:** 1 Hanusch Hospital, Vienna, Austria; 2 Krems an der Donau, Danube Private University, Krems an der Donau, Austria; 3 Lower Austrian Centre for Rheumatology, State Hospital Korneuburg-Stockerau, Stockerau, Austria; 4 Department of Rheumatology, Hospital of Bruneck, Teaching Hospital of the Paracelsius Medical University, Bruneck, Italy; 5 Rheumatologie im Struenseehaus, Hamburg, Germany

**Keywords:** care structure, FTE, RheumaFacts, rheumatology workforce, supply

## Abstract

**Background:**

The demand for rheumatologic specialist care is rising across Europe, driven by aging populations, earlier diagnoses, and increasingly complex treatment regimens as well as increased complexity of patients, e.g., multi-morbidity. At the same time, the speciality faces growing workforce shortages. This study aims to provide a first cross-national snapshot of rheumatology workforce supply and care structures in Europe.

**Methods:**

A structured questionnaire was distributed to the members of the Section of Rheumatology of the UEMS (Union Européenne des Médecins Spécialistes). The survey, conducted in 2021 and updated in 2023, assessed specialist numbers, work allocation, care settings, and consultation patterns across 17 European countries.

**Results:**

Data from 24 respondents representing 17 countries revealed substantial differences in rheumatology care organization. While some countries rely predominantly on hospital outpatient clinics, others favor private practice models. The number of rheumatologists ranged from 0.7 to 5.1 per 100,000 inhabitants, with marked variation in full-time equivalents (FTEs), part-time work, and gender distribution. Non-clinical duties such as administration, teaching, and research consume up to 40% of working time in some settings. Non-inflammatory musculoskeletal conditions account for approximately 23% of rheumatology consultations, though their management varies widely between countries.

**Conclusion:**

This survey underscores the heterogeneity of rheumatologic care across Europe, both in workforce availability and in healthcare delivery models. The data highlight the need for context-specific workforce planning and serve as a preliminary contribution to the broader EULAR initiative “RheumaFacts,” which aims to establish a standardized, comparative database on rheumatology workforce, demand, and need.

## Introduction

Providing specialists for patients with rheumatic and musculoskeletal diseases (RMDs) faces multifactorial challenges, among which the following are most important: (1) increased demand for rheumatologic service due to increasing life expectancy in the general population and the resulting higher prevalence of RMDs (1–4), (2) earlier referrals due to new classification/diagnostic criteria (5, 6), (3) improved patient survival resulting from new therapeutic options (7–10), (4) growing need for specialized expertise to monitor these treatments, especially regarding side effects and comorbidities (11–13), (5) increased complexity of patients due to multi-morbidity (6) an increasing number of follow-up visits required by treat-to-target strategies (14) (7) a shrinking workforce due to the retirement of the baby boomer generation within the next decade (15–18), (8) additional shortages owing to the rise of part-time work among younger rheumatologists (9) difficulties in recruiting new rheumatologists.

Despite the urgency of this issue, only a few studies have attempted workforce projections in rheumatology. These projections vary significantly between different countries, which complicates the establishment of a reliable foundation for policy decisions (19–23).

Systematic literature reviews conducted in 2016 and 2018 aimed to synthesize data on workforce needs, leading the EULAR (former *European League against Rheumatim*, now *European Alliance of Associations for Rheumatology*) to develop 10 “Points to Consider” for conducting such studies. These address aspects such as supply, demand, and needs for care in diverse geopolitical contexts, incorporating future and demographic considerations (24, 25). In 2023, EULAR launched “RheumaFacts”—an initiative to collect structured data on RMDs and their impact on individuals, society, and healthcare systems across all EULAR member countries. This initiative combines data from open sources and member societies, enabling systematic comparisons of rheumatologic workforce, demand, and care provision across Europe (26).

To gain a preliminary understanding of the current supply of rheumatology specialist care across Europe, we conducted a survey among the members of the Section of Rheumatology of the UEMS. The findings of this survey are presented below.

## Materials and methods

### Participants

All 57 delegates representing 30 UEMS member states were informed about the survey and invited to participate during a general assembly of the section in 2021. The questionnaire was subsequently sent via email, with two reminders issued to non-respondents.

National delegates are selected by the most representative non-governmental organization representing specialist medical doctors in rheumatology of a European Union Member State, usually the national societies for rheumatology of the different countries. The data provided are based on expert estimates together with official data if present.

### Questionnaire

The survey instrument was adapted from the study by Puchner et al. on the rheumatologic workforce in Austria (20). It collected data on working hours, division of responsibilities, and numbers of rheumatologists in each country (see Supplementary material 1).

### Topics included

- Distribution of care responsibilities among specialties (e.g., rheumatology, internal medicine, rehabilitation),
- Number of rheumatologists (ideally in full-time equivalents, FTE) and their theoretical working hours,
- Patient numbers per week, including proportions of patients with inflammatory versus non-inflammatory RMDs,
- Average time spent on initial and follow-up visits, and the frequency of follow-ups per patient per year.

The survey was conducted in 2021 and updated in 2023.

### Demographic data

Population figures for the 17 participating countries were obtained from the website of the United Nations Department of Economic and Social Affairs, Population Division (27). The numbers of rheumatologists per country were provided by the participants and double checked with the numbers the UEMS receives by the national societies.

### Statistics

Descriptive statistics were used to summarize the data, including mean, median, range, standard deviation, and interquartile range (IQR). Results are presented as mean ± SD for continuous variables and as median with IQR for ordinal data.

## Results

### Response rate and demographics

A total of 24 out of 57 delegates (9 female, 15 male) from 17 countries responded, yielding a 42% response rate. Work settings among the delegates included university hospitals (*n* = 17), community hospitals (*n* = 5), and private practices (*n* = 3). Responses were received from: Austria, Belgium, Finland, Germany, Greece, Hungary, Italy, Poland, Portugal, Slovakia, Slovenia, Spain, Sweden, Switzerland, the Netherlands, Turkey, and the UK.

### Responsibilities and duties of rheumatologists in different countries

The responsibility for managing patients with RMDs (including non-inflammatory RMDs) varies considerably across countries. In Hungary, almost only rheumatologists are responsible for these patients, whereas in other countries, care is also provided by orthopedic surgeons and general practitioners. Additional specialties involved include internal medicine, physical medicine, rehabilitation, sports medicine, neurology, neurosurgery, endocrinology, and pain medicine.

**Figure 1:**
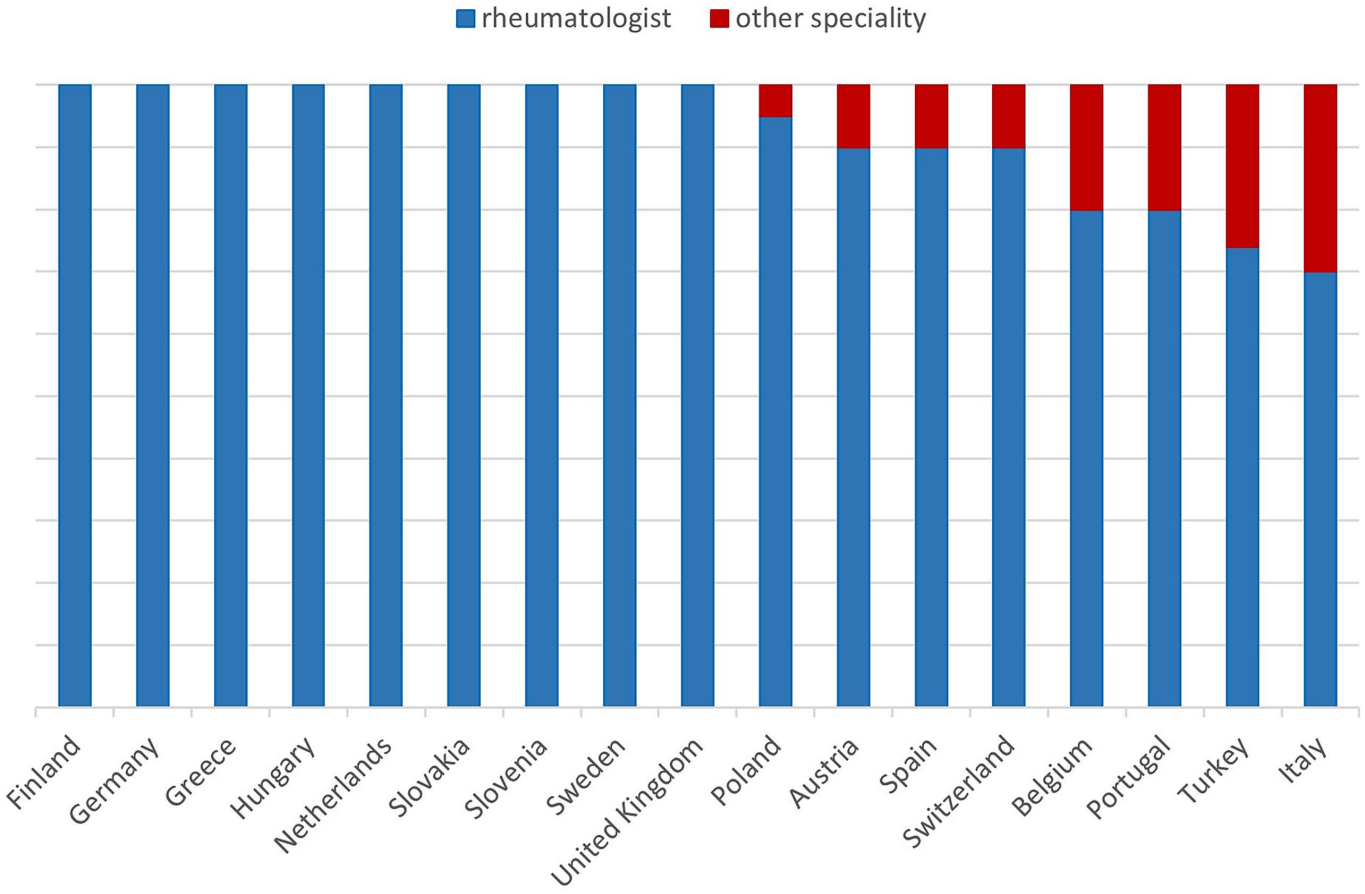
Specialists treating patients with inflammatory rheumatic diseases in different countries. Other specialities: general practitioner, orthopedic surgeon, internal medicine, rehabilitation specialist, sports doctor, neurologist, endocrinologist. Finland: Diagnosis and start of therapy rheumatologist, follow-up care shared by rheumatologist and general practitioner.

Inflammatory RMDs are exclusively managed by rheumatologists in eight countries, including Germany and the UK (Figure 1). In others, up to 25% of these patients are seen by other specialists. Finland uses a shared-care model, where diagnosis and initiation of therapy are performed by rheumatologists, while follow-up care is shared with general practitioners.

The proportion of non-inflammatory RMD patients seen by rheumatologists ranges from 10 to 65%, with the lowest values in Finland and Germany and the highest in Greece, Hungary, Turkey, and the UK. Overall, about 23% of patients treated by rheumatologists have non-inflammatory conditions.

### Care settings for inflammatory RMDs

**Figure 2:**
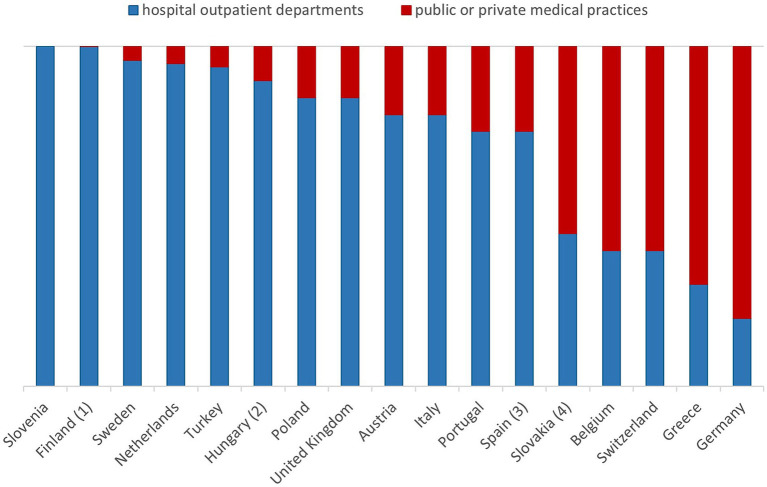
Institutions caring for patients with inflammatory rheumatic diseases in different countries. (1): Later on general practitioners with treatment paths for already diagnosed patients. (2): Including 40% in outpatient clinics. (3): Hospitals also including outpatient clinics, with private health insurance in private practices. (4): About 10% of patients are taken care of by both.

In 12 of 17 countries, over 75% of patients with inflammatory RMDs are managed in hospital outpatient clinics. This figure reaches nearly 100% in Slovenia, Sweden, Turkey, and the Netherlands. Also in Finland nearly all patients are seen by a rheumatologist in hospital outpatient clinics at the beginning of the disease, but later on are also managed by general practitioners according to treatment paths. In contrast, in Germany and Greece, most care is provided through public and private practices. Spain shows a dual system: public insurance patients are treated in outpatient clinics, while privately insured patients often receive care in private practices (Figure 2).

### Working hours and duties

**Figure 3:**
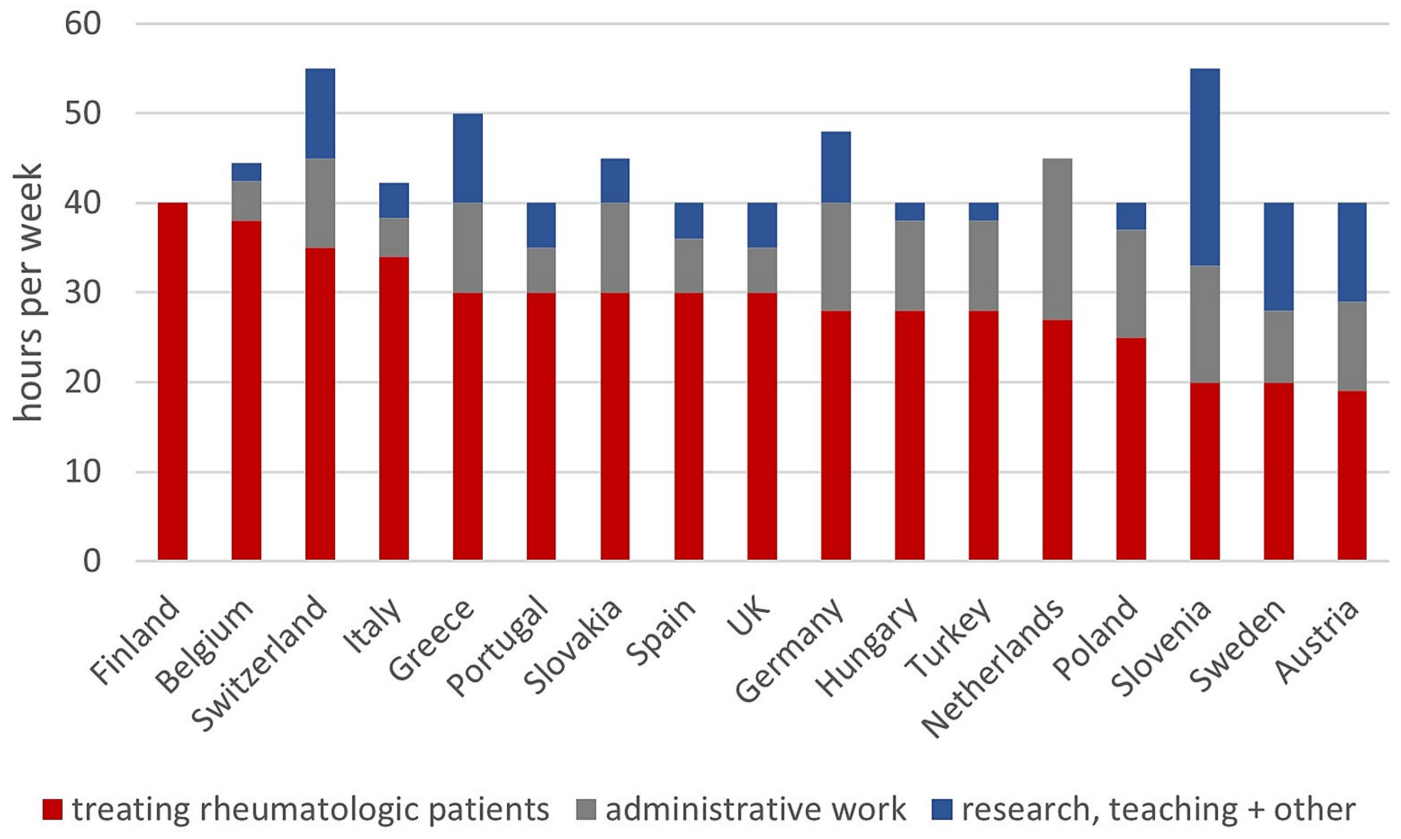
Working hours and duties of rheumatologists in different countries (in hours per week).

Reported FTEs range from 40 to 55 working hours per week. The proportion of time dedicated to direct care of rheumatologic patients ranges from 36 to 100%, with Finland reporting the highest. In 10 countries, more than 25% of time is allocated to administrative duties. Austrian rheumatologists spend 20% of their time treating general internal medicine patients (Figure 3). In most countries time spent on teaching and research varies between different hospital types (academic versus non-academic clinics) (data not shown).

### Time allocated to first appointments, follow-ups, and numbers of patients seen per week

**Figure 4:**
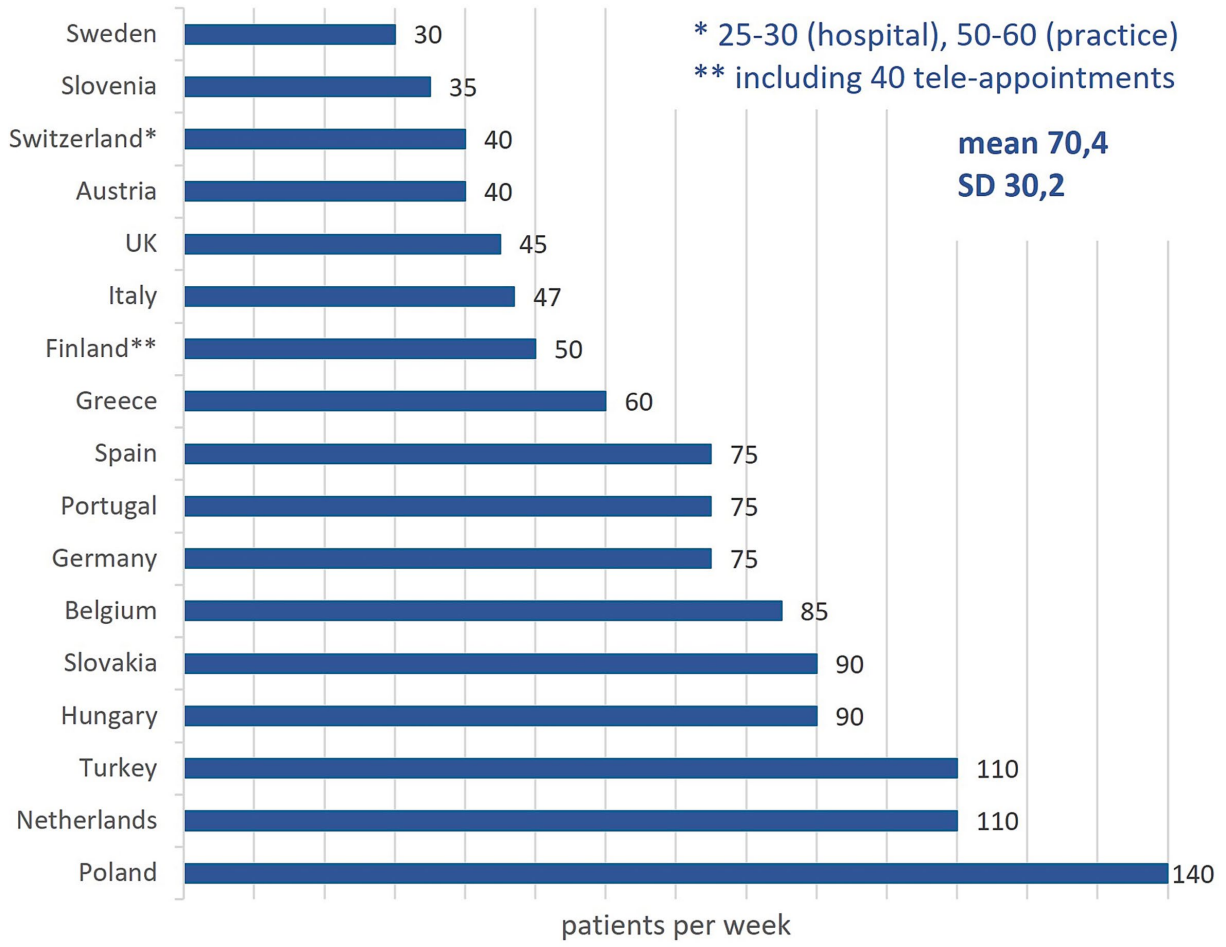
Number of patients a rheumatologist sees per week in different countries.

The average number of patients seen per rheumatologist per week is 70.4 (SD: 30.2), ranging from 30 in Sweden to 140 in Poland. Finland includes teleconsultations in this figure, while Switzerland reports differing volumes between hospitals and private practice (Figure 4).

First consultations typically last 30 min, with extremes ranging from 15 min in Turkey to 60 in Sweden and Switzerland. Follow-up visits for inflammatory RMDs last 15–25 min on average, with Finland reporting up to 45 min. For non-inflammatory RMDs, follow-up times are similar ranging between 10 and 25 min, although in Finland, Germany, and Sweden these patients are not routinely seen.

**Figure 5:**
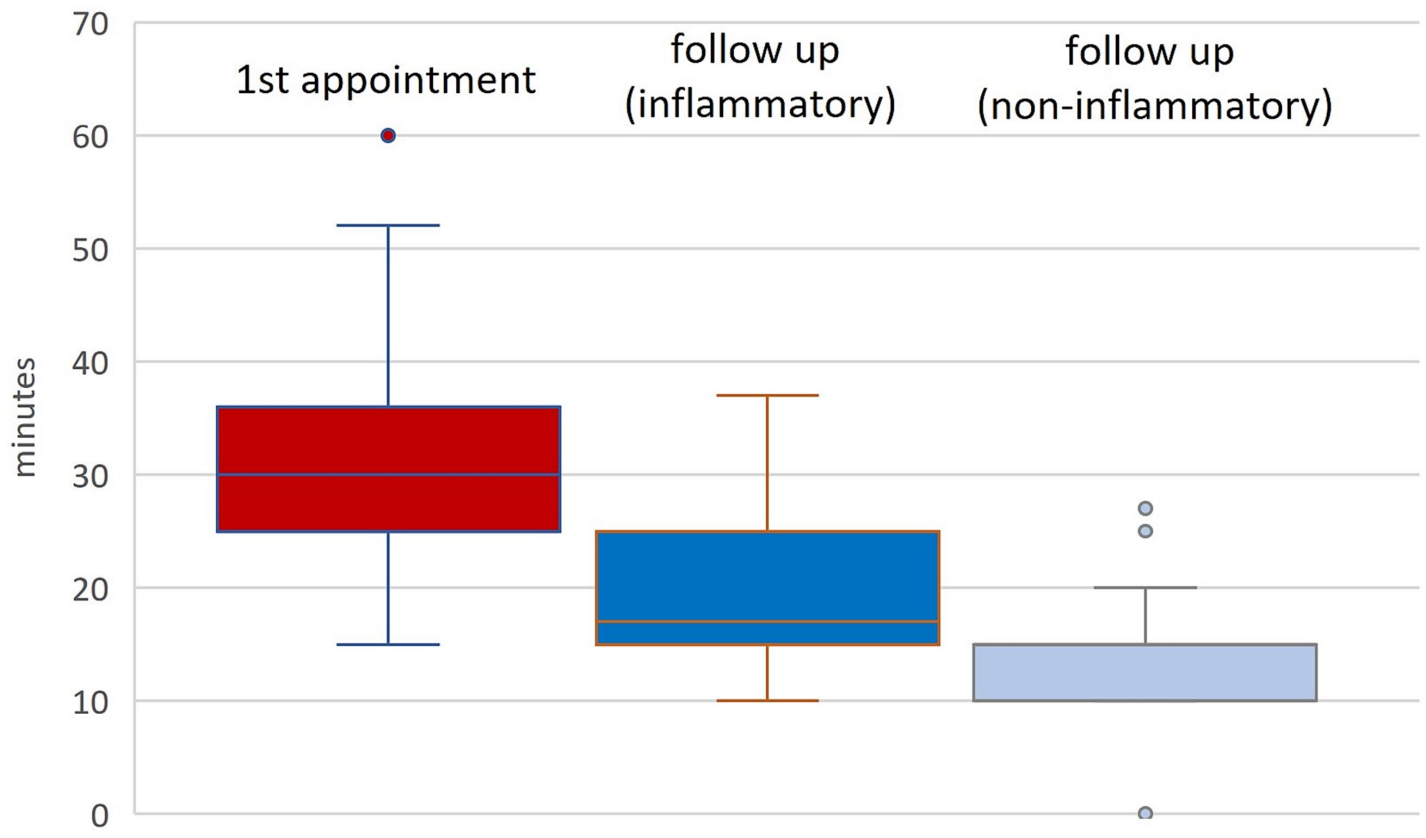
Time allocated to 1st appointments and follow up visits (in minutes) in different countries.

Follow-up intervals for inflammatory RMDs vary from 1 to 12 months, with most countries aiming for 3–4 visits annually. Non-inflammatory cases are typically seen yearly, except in Hungary and Turkey, where they are reviewed every 3 months (Figure 5).

### Number of rheumatologists per population

Countries reported the number of rheumatologists, with an effort to adjust for FTEs. In cases where exact part-time information was not provided, a 75% FTE assumption was used as a standard estimate for part-time roles (Table 1).

**Table 1:** Numbers of rheumatologists and inhabitants in the different countries.

Country	Rheumatologists (n)	Inhabitants § (Mio.)	Rheumatologists per 100,000	Full time (n)	Part time (n)	FTE* (n)	FTE per 100,000
Austria	299	9.14	3.3	215	84	278	3
Belgium	279	11.7	2.4	−	−	175	1.5
Finland	100	5.59	1.8	90	10	97	1.7
Germany	1,164	84.4	1.4	700	464	1,048	1.3
Greece	372	10.35	3.6	279	93	349	3.4
Hungary	300	9.68	3.1	400	0	300	3.1
Italy	1,614	59.56	2.7	1,050	564	1,474	2.5
Netherlands	352	18.02	2	−	−	−	−
Poland	1,250	38.81	3.2	1,000	250	1,188	3.1
Portugal	247	10.43	2.4	−	−	−	−
Slovakia	95	5.52	1.7	85	10	93	1.7
Slovenia	31	2.12	1.5	−	−	−	−
Spain	1,600	47.91	3.3	−	−	−	−
Sweden	289	10.52	2.7	−	−	189	1.8
Switzerland	450	8.84	5.1	225	225	394	4.5
Turkey	650	87.17	0.7	−	−	−	−
UK	700	68.44	1	466	233	640	0.9

*Calculated with 75% part time if not otherwise specified. §Source: United Nations Department of Economic and Social Affairs, Population Division (2023). FTE, full time equivalent.

**Figure 6:**
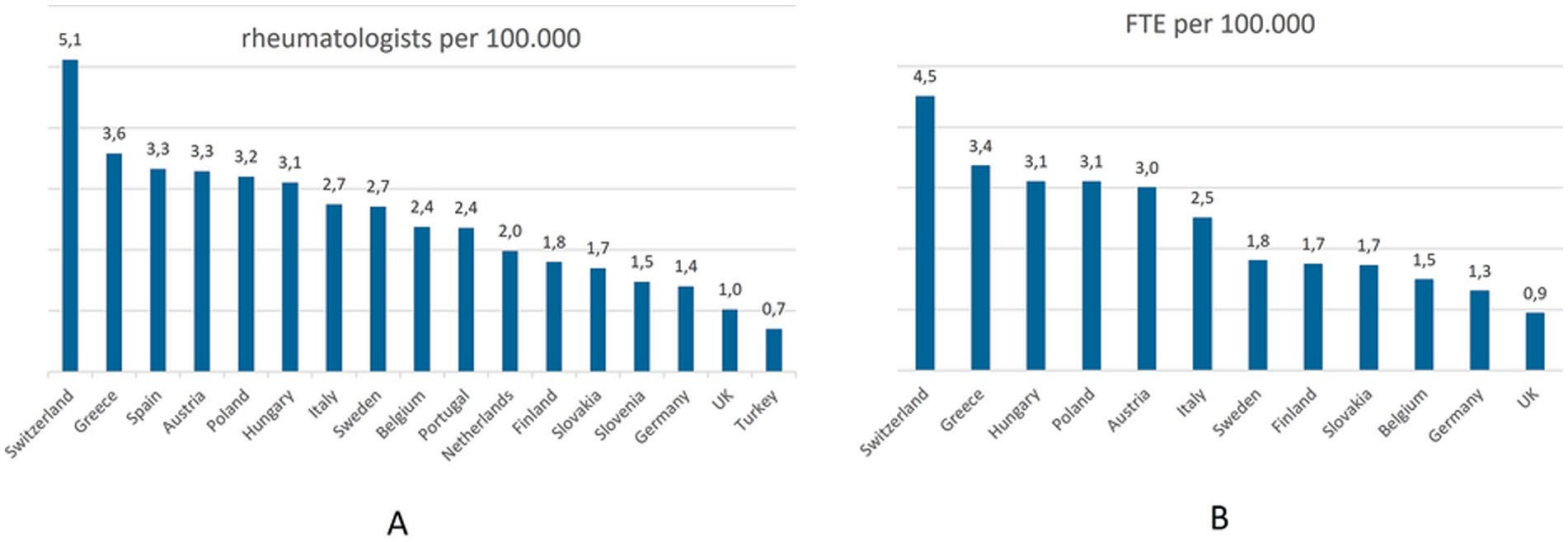
**(A,B)** Rheumatologists and FTEs per 100,000 inhabitants.

Rheumatologist density ranges from 0.7 to 5.1 per 100,000 inhabitants (mean: 2.5/100,000; SD: 1.1). The highest numbers were found in Switzerland and Greece; the lowest in Turkey, Germany, and the UK (Table 1 and Figure 6A) Adjusted FTE densities reflect these results (Figure 6B).

Due to lack of data regarding part time work in some countries the count of physicians may overestimate the actual clinical capacity.

**Figure 7:**
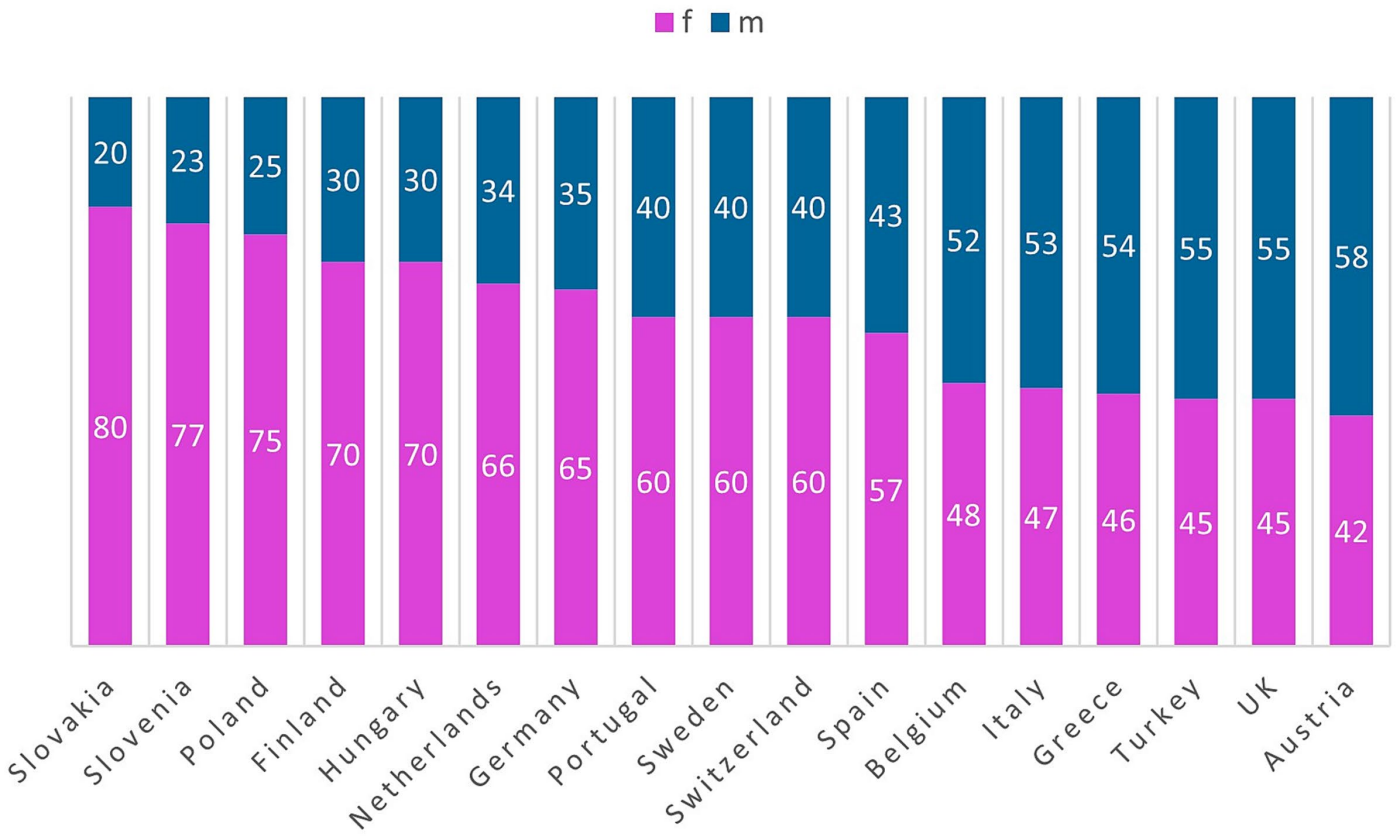
Gender distribution in the different countries.

Female representation among rheumatologists varies widely, from 42% (Austria) to 80% (Slovakia) (Figure 7).

## Discussion

This survey provides an overview of the current status of rheumatologic specialist care across 17 European countries, highlighting striking variations in workforce distribution, organizational structures, and care responsibilities.

One key finding is the substantial heterogeneity in the organization of rheumatologic care, particularly between hospital-based and private practice models. While countries like Sweden and Slovenia deliver care almost exclusively through hospital outpatient departments, others such as Germany and Greece rely predominantly on privately practicing rheumatologists. These differences might reflect not only national healthcare policies but also historical and economic frameworks, underscoring the need for context-specific workforce planning considering regional differences.

Another significant observation is the wide variation in the number of rheumatologists per 100,000 inhabitants, ranging from fewer than 1 in Turkey to more than 4 in Switzerland. This discrepancy is further compounded by differences in working hours, distribution of duties, and administrative burden, with some countries reporting that over 25% of working time is spent on non-clinical tasks such as documentation, teaching, or research. In contrast, others (e.g., Finland) report nearly exclusive focus on patient care.

The increasing trend toward part-time work, especially among younger rheumatologists, poses an additional challenge for workforce planning. While part-time work can help address work–life balance, it also reduces the effective full-time equivalent (FTE) capacity and may amplify future shortages as current full-time specialists retire. Notably, many rheumatologists initially work full time during their training period but reduce their working hours after achieving board certification. This pattern further limits the number of rheumatologists available for direct patient care and exacerbates the gap between nominal and effective workforce capacity. This trend is accompanied by a growing proportion of female rheumatologists, with some countries reporting over 70% female representation. This demographic shift necessitates flexible, gender-sensitive workforce policies that ensure sustainable specialist care without compromising service delivery.

Another pivotal factor obscuring inter-regional workforce planning is the medical migration from countries with lower socioeconomic levels to higher income countries, worsening the situation for those countries that are losing specialized personnel.

To ensure good rheumatological care in the future, national and international societies must focus on recruiting young physicians with an interest in the field and ensure high-quality training as well as good working conditions. Rheumatologic summer schools for students seem to be an adequate instrument to attract young professionals (28).

Our findings also highlight differences in the scope of duties across countries. In some healthcare systems, rheumatologists share responsibilities with general practitioners or specialists in physical medicine, neurology, or orthopedics, particularly in the management of non-inflammatory musculoskeletal disorders. While this interdisciplinary approach may enhance access to care, it can also obscure the delineation of responsibilities and complicate workforce estimation. In our study we did not ask for the presence of non-physician health care professionals reducing the workload for rheumatologists, this aspect should be addressed in further studies.

Importantly, this survey reveals that non-inflammatory rheumatic diseases account for approximately one-quarter of all rheumatology consultations, although this proportion varies considerably. The role of rheumatologists in managing these conditions is not uniformly defined across countries, which may reflect differing models of care or resource constraints.

Several limitations should be acknowledged. The results are based in part on estimations provided by national delegates, which introduces a degree of subjectivity and potential bias. In addition, the response rate was relatively low (42%), which may limit the representativeness of the findings. The substantial diversity of healthcare settings—including differences between urban and rural areas, hospital types, and reimbursement systems—also reduces data comparability. Furthermore, national differences in care organization, such as shared care models, insurance frameworks, the scope of rheumatologists’ responsibilities, and the level of support from other health care professionals, complicate direct cross-country comparisons. Moreover, socioeconomic differences between countries arising from interregional economic disparities, which also influence healthcare workforce planning, represent an additional limitation of this study.

This project serves as a preliminary exploration into the European rheumatology workforce. It is intended as an appetizer to the ongoing EULAR initiative “RheumaFacts,” which aims to collect structured data from open sources and national societies. This larger initiative will enable systematic and comparable assessments of workforce supply, demand, and needs across Europe, offering a stronger foundation for future health policy and resource allocation.

## Data Availability

The original contributions presented in the study are included in the article/Supplementary material, further inquiries can be directed to the corresponding author.

## References

[1] Briggs AM Cross MJ Hoy DG Sànchez-Riera L Blyth FM Woolf AD . Musculoskeletal health conditions represent a global threat to healthy aging: a report for the 2015 World Health Organization world report on ageing and health. Gerontologist. (2016) 56 Suppl 2:S243–55. doi: 10.1093/geront/gnw002, 26994264

[2] Smith E Hoy DG Cross M Vos T Naghavi M Buchbinder R . The global burden of other musculoskeletal disorders: estimates from the global burden of disease 2010 study. Ann Rheum Dis. (2014) 73:1462–9. doi: 10.1136/annrheumdis-2013-204680, 24590181

[3] Vos T Flaxman AD Naghavi M Lozano R Michaud C Ezzati M . Years lived with disability (YLDs) for 1160 sequelae of 289 diseases and injuries 1990-2010: a systematic analysis for the global burden of disease study 2010. Lancet. (2012) 380:2163–96. doi: 10.1016/S0140-6736(12)61729-2, 23245607 PMC6350784

[4] Hootman JM Helmick CG Barbour KE Theis KA Boring MA. Updated projected prevalence of self-reported doctor-diagnosed arthritis and arthritis-attributable activity limitation among US adults, 2015-2040. Arthritis Rheumatol. (2016) 68:1582–7. doi: 10.1002/art.39692, 27015600 PMC6059375

[5] Radner H Neogi T Smolen JS Aletaha D. Performance of the 2010 ACR/EULAR classification criteria for rheumatoid arthritis: a systematic literature review. Ann Rheum Dis. (2014) 73:114–23. doi: 10.1136/annrheumdis-2013-203284, 23592710

[6] Emery P Breedveld FC Dougados M Kalden JR Schiff MH Smolen JS. Early referral recommendation for newly diagnosed rheumatoid arthritis: evidence based development of a clinical guide. Ann Rheum Dis. (2002) 61:290–7. doi: 10.1136/ard.61.4.290, 11874828 PMC1754044

[7] Chiu YM Lu YP Lan JL Chen DY Wang JD. Lifetime risks, life expectancy, and health care expenditures for rheumatoid arthritis: a Nationwide cohort followed up from 2003 to 2016. Arthritis Rheumatol. (2021) 73:750–8. doi: 10.1002/art.41597, 33295139 PMC8247851

[8] Almutairi KB Inderjeeth CA Preen DB Keen HI Nossent JC. Mortality trends among patients with rheumatoid arthritis in Western Australia. Rheumatol Ther. (2023) 10:1021–37. doi: 10.1007/s40744-023-00562-0, 37335433 PMC10326173

[9] Dadoun S Zeboulon-Ktorza N Combescure C Elhai M Rozenberg S Gossec L . Mortality in rheumatoid arthritis over the last fifty years: systematic review and meta-analysis. Joint Bone Spine. (2013) 80:29–33. doi: 10.1016/j.jbspin.2012.02.005, 22459416

[10] Yen EY Shaheen M Woo JMP Mercer N Li N McCurdy DK . 46-year trends in systemic lupus erythematosus mortality in the United States, 1968 to 2013: a Nationwide population-based study. Ann Intern Med. (2017) 167:777–85. doi: 10.7326/M17-0102, 29086801 PMC6188647

[11] Smolen JS Landewé RBM Bergstra SA Kerschbaumer A Sepriano A Aletaha D . EULAR recommendations for the management of rheumatoid arthritis with synthetic and biological disease-modifying antirheumatic drugs: 2022 update. Ann Rheum Dis. (2023) 82:107–18. doi: 10.1136/ard-2022-223356, 36357155

[12] Gossec L Baraliakos X Kerschbaumer A de Wit M McInnes I Dougados M . EULAR recommendations for the management of psoriatic arthritis with pharmacological therapies: 2019 update. Ann Rheum Dis. (2020) 79:700–12. doi: 10.1136/annrheumdis-2020-217159, 32434812 PMC7286048

[13] Ramiro S Nikiphorou E Sepriano A Ortolan A Webers C Baraliakos X . ASAS-EULAR recommendations for the management of axial spondyloarthritis: 2022 update. Ann Rheum Dis. (2023) 82:19–34. doi: 10.1136/ard-2022-223296, 36270658

[14] Smolen JS Breedveld FC Burmester GR Bykerk V Dougados M Emery P . Treating rheumatoid arthritis to target: 2014 update of the recommendations of an international task force. Ann Rheum Dis. (2016) 75:3–15. doi: 10.1136/annrheumdis-2015-207524, 25969430 PMC4717393

[15] Miloslavsky EM Bolster MB. Addressing the rheumatology workforce shortage: a multifaceted approach. Semin Arthritis Rheum. (2020) 50:791–6. doi: 10.1016/j.semarthrit.2020.05.009, 32540672 PMC7255118

[16] Kilian A Upton LA Battafarano DF Monrad SU. Workforce trends in rheumatology. Rheum Dis Clin N Am. (2019) 45:13–26. doi: 10.1016/j.rdc.2018.09.002, 30447742

[17] Lee CU Kim JN Kim JW Park SH Lee H Kim SK . Korean rheumatology workforce from 1992 to 2015: current status and future demand. Korean J Intern Med. (2019) 34:660–8. doi: 10.3904/kjim.2016.417, 29232941 PMC6506748

[18] Widdifield J Bernatsky S Pope JE Kuriya B Barber CEH Eder L . Evaluation of rheumatology workforce supply changes in Ontario, Canada, from 2000 to 2030. Healthc Policy. (2021) 16:119–34. doi: 10.12927/hcpol.2021.26428, 33720829 PMC7957360

[19] Dejaco C Lackner A Buttgereit F Matteson EL Narath M Sprenger M. Rheumatology workforce planning in Western countries: a systematic literature review. Arthritis Care Res. (2016) 68:1874–82. doi: 10.1002/acr.22894, 27015436

[20] Puchner R Vavrovsky A Pieringer H Hochreiter R Machold KP. The supply of rheumatology specialist Care in Real Life. Results of a Nationwide survey and analysis of supply and needs. Front Med. (2020) 7:16. doi: 10.3389/fmed.2020.00016, 32083088 PMC7002545

[21] Brophy J Marshall DA Badley EM Hanly JG Averns H Ellsworth J . Measuring the rheumatology workforce in Canada: a literature review. J Rheumatol. (2016) 43:1121–9. doi: 10.3899/jrheum.151174, 27036382

[22] Harrison MJ Lee S Deighton C Symmons DP. UK rheumatology consultant workforce provision 2007-9: results from the BSR/Arthritis Research UK consultant workforce register. Clin Med. (2011) 11:119–24. doi: 10.7861/clinmedicine.11-2-119, 21526690 PMC5922730

[23] Zink A Braun J Gromnica-Ihle E Krause D Lakomek HJ Mau W . Memorandum der Deutschen Gesellschaft für Rheumatologie zur Versorgungsqualität in der Rheumatologie – update 2016 [memorandum of the German Society for Rheumatology on the quality of treatment in rheumatology - update 2016]. Z Rheumatol. (2017) 76:195–207 German. doi: 10.1007/s00393-017-0297-1, 28364218

[24] Unger J Putrik P Buttgereit F Aletaha D Bianchi G Bijlsma JWJ . Workforce requirements in rheumatology: a systematic literature review informing the development of a workforce prediction risk of bias tool and the EULAR points to consider. RMD Open. (2018) 4:e000756. doi: 10.1136/rmdopen-2018-000756, 30714580 PMC6336097

[25] Dejaco C Putrik P Unger J Aletaha D Bianchi G Bijlsma JW . EULAR 'points to consider' for the conduction of workforce requirement studies in rheumatology. RMD Open. (2018) 4:e000780. doi: 10.1136/rmdopen-2018-000780, 30714579 PMC6336096

[26] EULAR. Available online at: https://www.eular.org/eular-rheumafacts (accessed January 12, 2026)

[27] United Nations, Department of Economic and Social Affairs, Population Division. Available online at: https://population.un.org/ (accessed January 12, 2026)

[28] Sautner J Puchner R Reisch M Alkin A Duftner C Dejaco C. Professional development is the key to securing a future rheumatology workforce. Long term evaluation of a summer school for medical students-a national scientific society's educational initiative. Front Med. (2024) 11:1413544. doi: 10.3389/fmed.2024.1413544, 39296892 PMC11409003

