# Supplementary material for: Rheumatology specialist care in Europe: workforce trends and regional variations from a UEMS survey (European Union of medical specialists)
Source: Front Med (Lausanne). 2026 Feb 24;13:1786042. doi: 10.3389/fmed.2026.1786042 (PMC12971878; doi:10.3389/fmed.2026.1786042)
Supplement: Supplementary file 1 [file Table_1.docx]

The Supply of Rheumatology Specialist Care in Real Life

Questionnaire for Members of the European Board of Rheumatologists (UEMS)

Dear Colleagues,

as members of the European Board of Rheumatologists (UEMS), we are kindly requesting your participation in this project. Once you have completed this questionnaire, we shall proceed to compare the situation and workload in different European countries. This will be based on the way you understand and practice rheumatology in your given country.

We kindly ask you not to answer from personal experience, but to imagine what answer would be given by most of your colleagues in your country (a kind of representative vote).

1. In which country do you practice rheumatology? ...............................

2. Which specialists treat adult patients with musculoskeletal diseases in your country?

- Rheumatologists o
- Orthopaedic surgeons o
- General practitioners o
- Other specialists

3. Which specialists treat patients with inflammatory rheumatic diseases?

Only internal rheumatologists: Yes o No o

If you answered no, please select and indicate in percentages

- Internal rheumatologists o ( ...............%)
- Specialists in physical medicine o (...............%)
- Other specialists .............................. o (….......….%) (please estimate percentage for all)

4. What institutions see and treat patients with inflammatory rheumatic diseases?

- hospital outpatient departments o (….......… %)
- private practices o (………… **%**)

5. How many practising rheumatologists work in your country? ..........................

- If possible please specify the distribution of full time, part time or full time equivalent:

…………………………………………………………..

6. Where do these rheumatologists work?

- University clinics o (….......… %)
- Hospitals o (….......… %)
- Office based/private practices o (….......… %)

If mixed please comment:…………………………………………………………….

7. What is the percentage of female rheumatologists in your country? ...............%

Please answer the following questions with the aim of giving representative answers which might be true for the majority of rheumatologists in your country:

8. How many hours per week does a rheumatologist work (as a physician) in total?

(this should include general patient care, night duty, administrative work, research, teaching etc.)?

………………………………

9. How many of the hours per week specified above, does a rheumatologist work:

- treating purely rheumatological patients? ……………………….
- doing administrative work? ……………………….
- doing research and teaching? ……………………….
- Other ……………………….

10. How many patients does a rheumatologist see per week? ...............

11. How much time is allocated to the first appointment with a new patient? ............minutes

12. How much time is allocated to a follow-up appointment with a patient with inflammatory or autoimmune diseases? ..........minutes

13. How much time is allocated to a follow-up appointment with a patient with non- inflammatory musculoskeletal diseases? ........... minutes

14. What is the percentage of patients with non-inflammatory musculoskeletal diseases seen by a rheumatologist in his or her consulting hours? .........…%

15. Within what period of time will patients with inflammatory rheumatic diseases generally be seen for follow-up appointments? ............ months

16. Within what period of time will patients with non-inflammatory musculoskeletal diseases generally be seen for follow-up appointments? ............ months

o no follow-up appointments
